# Key molecular mechanisms of mitochondrial metabolic pathways in specific cell subpopulations of pancreatic cancer based on scRNA-seq and bulk RNA-seq

**DOI:** 10.1371/journal.pone.0351701

**Published:** 2026-07-02

**Authors:** Jianping Liu, Tao Liu, Le Yang, Yi Hu, Chao Luo, Dan Wu, Xingjian Yang

**Affiliations:** 1 Department of General Surgery, The First People’s Hospital of Shuangliu District, Chengdu, China; 2 Department of Operating Room, The First People’s Hospital of Shuangliu District, Chengdu, China; The University of Sheffield, UNITED KINGDOM OF GREAT BRITAIN AND NORTHERN IRELAND

## Abstract

**Background:**

Pancreatic cancer (PC) is a highly malignant tumor with increasing incidence, mortality, and a low five-year survival rate. Mitochondrial metabolic reprogramming plays a crucial role in tumor development, but the molecular mechanisms in different cell subpopulations of PC remain unclear. This study aims to integrate single-cell RNA sequencing (scRNA-seq) and bulk RNA-seq to explore mitochondrial metabolism in PC.

**Methods:**

Transcriptome datasets (GSE183795, GSE16515, GSE197177, and TCGA-PAAD) were downloaded from the GEO and TCGA databases. Differentially expressed genes (DEGs) were identified using the limma package for bulk RNA-seq and the Seurat package for scRNA-seq. Weighted gene co-expression network analysis (WGCNA) was performed to identify key modules and hub genes. Machine learning algorithms screened key genes, and functional enrichment analysis was conducted using clusterProfiler. PPI, ceRNA, and transcription factor networks explored gene regulation. Immune infiltration analysis, drug prediction, and molecular docking were conducted on key genes. The prognostic value of the key genes was evaluated using clinical data from TCGA.

**Results:**

A total of 1238 bulk RNA-DEGs and 8231 scRNA-DEGs in fibroblasts were identified. Integration of both datasets revealed 536 DEGs. WGCNA identified 6 modules associated with PC. By intersecting DEGs, hub genes, and mitochondrial metabolism-related genes, 18 candidate genes were obtained. These genes were enriched in glucose metabolism and mitochondrial outer membrane pathways. Three key genes (IFI27, PKM, and RSAD2) were selected using machine learning. PPI, ceRNA, and transcription factor networks provided regulatory insights. Immune infiltration analysis showed significant differences in immune cells, particularly in T cells CD4 memory resting and macrophages M2. Drug prediction and molecular docking identified potential drugs for these genes. Survival analysis indicated that high expression of these genes correlated with poor prognosis.

**Conclusion:**

This study integrates scRNA-seq and bulk RNA-seq data to identify three key genes (IFI27, PKM, and RSAD2) and their immune-related mechanisms in PC. These findings offer new insights into the pathogenesis and potential therapeutic targets for PC.

## 1 Introduction

Pancreatic cancer (PC) is a malignant tumor with high incidence and mortality rates globally. Statistics show that in 2020, there were approximately 496,000 new cases of PC and 466,000 deaths worldwide, with both incidence and mortality rates showing an increasing trend each year [1]. PC exhibits significant heterogeneity, leading to varied clinical presentations and prognoses [2]. Currently, main treatment modalities for PC include surgery, chemotherapy, radiotherapy, targeted therapy, and combination therapies; however, the overall efficacy remains limited, with a typical survival period of less than 5 years [3,4]. Therefore, delving deeper into the pathogenesis of PC, and identifying new therapeutic targets and strategies, holds significant importance in improving the prognosis of PC patients.

The tumor microenvironment (TME) is a key determinant of PC progression. The PC microenvironment is a dynamic and complex system comprising various cell types such as fibroblasts, immune cells, endothelial cells, etc. These cells, through interactions with cancer cells and the extracellular matrix, collectively regulate the initiation, development, and metastasis of PC [5]. Among these, immune cells play a crucial role in the progression of PC. Studies have shown that immune cells infiltrating PC tissues mainly include tumor-associated macrophages (TAMs), myeloid-derived suppressor cells (MDSCs), and regulatory T cells (Tregs), among others. These immunosuppressive cells can inhibit the body's anti-tumor immune response through mechanisms like secretion of immunosuppressive factors and induction of T cell exhaustion, promoting immune evasion and progression of PC [6]. Furthermore, CD8 + cytotoxic T lymphocytes (CTLs) as the main force in the body's anti-tumor immune response, their infiltration levels are closely related to the prognosis of PC patients, but often exist in a dysfunctional state within the PC microenvironment [7]. Importantly, checkpoint molecules (PD-L1, CTLA-4) in immune cells correlate with clinical outcomes, highlighting their prognostic value [8,9]. Therefore, exploring the heterogeneity and functional states of immune cells in the PC microenvironment is of significant importance in understanding the immune escape mechanisms of PC and developing new immune therapeutic strategies.

Importantly, fibroblasts, as a critical component of the PC microenvironment, also play a key role in the development of PC [10]. Research has found that fibroblasts in PC tissues exhibit an activated state and can promote the proliferation, invasion, and metastasis of PC cells through secretion of extracellular matrix, inflammatory factors, and growth factors [11]. Additionally, fibroblasts can reshape the metabolic microenvironment of PC by inducing processes like glycolysis and glutamine metabolism, providing nutritional support for the growth of PC cells [12]. Notabley, CAF-derived factors such as tenascin-C serve as independent prognostic markers correlating with poor survival [13]. Mitochondria, as the hub of cellular energy metabolism and signal transduction, are closely related to the activation of fibroblasts [14]. Studies suggest that mitochondrial metabolism reprogramming can regulate the activation phenotype of fibroblasts, participating in the progression of cancer, including PC [15,16]. Targeting the mitochondrial metabolism of fibroblasts in the PC microenvironment may serve as a new strategy for treating PC [17]. Meanwhile, alterations in mitochondrial metabolic genes represent potential therapeutic targets with prognostic significance. However, the molecular mechanisms underlying the promotion of PC development by fibroblasts currently lack in-depth and systematic understanding, necessitating further research.

Integrating single-cell RNA sequencing (scRNA-seq) and bulk RNA sequencing (bulk RNA-seq) is essential for comprehensive PC characterization [18]. scRNA-seq can depict the cellular composition and states of the tumor microenvironment at single-cell resolution, revealing interactions between different cell types [19]. However, scRNA-seq alone lacks population-level validation. Bulk RNA-seq provides comprehensive transcriptomic profiling across larger patient cohorts, enabling identification of gene signatures associated with clinical outcomes. Integrating both technologies bridges cellular mechanisms with clinical translation, allowing validation of single-cell findings and construction of robust prognostic models [20]. The Weighted Gene Co-expression Network Analysis (WGCNA) algorithm based on co-expression networks can cluster genes into different functional modules, identifying genes associated with phenotypes [21]. Moreover, machine learning algorithms can select the optimal combination of biomarkers from high-dimensional omics data to build disease prediction models [22]. Therefore, combining single-cell sequencing with bioinformatics analysis is expected to elucidate the molecular pathological mechanisms of PC at multiple levels.

In conclusion, this study integrates PC-related single-cell and bulk transcriptome datasets from public databases to analyze cellular heterogeneity and mitochondrial metabolism in the TME. By combining scRNA-seq for cellular characterization with bulk RNA-seq for clinical validation, we comprehensively investigate how immune cells and fibroblasts contribute to PC pathogenesis. Through differential expression analysis, WGCNA, and machine learning algorithms, we identify key prognostic genes and construct a risk prediction model. These genes will be evaluated for their prognostic value in predicting survival and therapeutic response, providing a molecular framework for patient stratification. Further analysis explores their biological functions, regulatory mechanisms, immune microenvironment relationships, and potential drug intervention strategies, providing new insights for PC diagnosis, prognosis assessment, and therapeutic development.

## 2 Methods

### 2.1 Data download and preprocessing

Transcriptome datasets related to PC were downloaded from the Gene Expression Omnibus (GEO) database and The Cancer Genome Atlas (TCGA) database, including GSE183795, GSE16515, and TCGA‑PAAD. The GSE183795 dataset was used as the training cohort and was generated on the GPL6244 platform (Affymetrix Human Gene 1.0 ST Array). It comprised 139 PC tissue samples and 105 adjacent non‑tumor and normal pancreatic tissue samples, all derived from patients with pancreatic ductal adenocarcinoma (PDAC) and normal pancreas.

The GSE16515 dataset served as an external validation cohort and was based on the GPL570 platform (Affymetrix Human Genome U133 Plus 2.0 Array). This dataset included 36 PC samples and 16 normal pancreatic samples, totaling 52 samples, among which 16 cases contained paired tumor and normal expression data, while 20 cases included tumor samples only. The TCGA‑PAAD dataset was used for survival analysis and consisted of 178 PC samples and 4 normal pancreatic samples. All datasets were preprocessed, including background correction, gene annotation, and data normalization.

To further investigate the cellular heterogeneity of PC, the single‑cell RNA sequencing dataset GSE197177, generated on the GPL18573 platform (Illumina NextSeq 500), was obtained from the GEO database. This dataset comprised single‑cell transcriptomic data from three primary untreated pancreatic ductal adenocarcinoma (PDAC) tumors, four liver metastases, and one adjacent normal pancreatic tissue sample. In the present study, single‑cell data from three primary untreated PDAC tumor samples and one adjacent normal pancreatic tissue sample were extracted for downstream analyses.Pancreatic ductal adenocarcinoma (PDAC) is the predominant histological subtype of PC; therefore, single‑cell RNA sequencing data obtained mainly from PDAC samples were used for downstream analyses. Single‑cell data processing was performed using the Seurat package, and high‑quality cells were retained based on the number of detected genes and the proportion of mitochondrial gene expression.

Furthermore, the human mitochondrial metabolism-related genes (MMGs) were obtained from the literature [23].

### 2.2 scRNA-seq single-cell data analysis

Based on the PC single-cell dataset GSE197177, quality control and preprocessing were performed using the Seurat package. The top 2000 highly variable genes were identified, and the top 10 highly variable genes were labeled. The data was normalized using the ScaleData function, followed by dimensionality reduction using Principal Component Analysis (PCA). The number of principal components for further analysis was determined based on PCA elbow plots and heatmaps. Subsequently, the cells were visualized and clustered using the Uniform Manifold Approximation and Projection (UMAP) algorithm for cell type annotation. Cell clusters were identified using the Shared Nearest Neighbor (SNN) graph and Louvain algorithm. Cell type annotation was conducted for each cell subpopulation using known cell type marker genes, and the expression of characteristic genes for each cell type was visualized using bubble plots. Cell type annotation for each cell subpopulation was determined by integrating relevant literature and databases. Additionally, the Wilcoxon test was employed to quantify the abundance of each cell type in the PC group and the normal group to understand the cellular composition of the tumor microenvironment. Specific cells related to mitochondrial metabolism in PC were identified through reported literature. Differential expression genes among different cells were analyzed using the FindMarkers function, and the differential expression genes were visualized. These genes of specific cells were referred to as single-cell differential expression genes (scRNA-DEGs) for subsequent analysis (P < 0.05 and |log_2_ fold change (FC)| > 0.5).

Importantly, although the single-cell dataset analyzed here has been reported previously, the original study was aimed at constructing a general single-cell atlas of PDAC; in contrast, the present work is specifically designed to dissect mitochondrial metabolic features within defined cell subpopulations and to integrate these single-cell observations with bulk transcriptomic profiling, WGCNA and machine learning. This cross-platform, mitochondrial-metabolism–oriented integration, which links cellular heterogeneity to clinically relevant prognostic genes, distinguishes our analysis from the original report and constitutes the main novelty of this study.

### 2.3 Transcriptome data differential expression analysis

The GSE183795 dataset was used as the training set for differential expression analysis using the limma package based on the threshold criteria of |log_2_ FC| > 0.5 and P < 0.05. The volcano plot was generated to visualize the distribution of up-regulated and down-regulated bulk RNA-DEGs, with the x-axis representing log2FC and the y-axis representing -log10(adjusted P-value). Heatmaps of the top differentially expressed genes were created using hierarchical clustering based on Euclidean distance and complete linkage method to reveal expression patterns across samples. The intersection of scRNA-DEGs and bulk RNA-DEGs was obtained to identify DEGs with consistent expression trends.

### 2.4 WGCNA

To identify co-expressed gene modules and hub genes associated with PC, we conducted WGCNA on the training dataset. Initially, the pickSoftThreshold function was used to determine the soft threshold power β by analyzing the scale-free fit index and average connectivity. The optimal soft threshold was selected to construct the adjacency matrix, which was then transformed into the Topological Overlap Matrix (TOM) to measure the co-expression similarity between genes. Subsequently, hierarchical clustering was performed on the TOM-based dissimilarity matrix, and the dynamic tree-cutting algorithm was employed to identify gene modules. The minimum module size was set to 30, and the cut height was set to 0.25. Module Eigengenes (MEs) were calculated as the first principal component of each module, representing the overall expression profile of the module. MEs were correlated with clinical features (tumor vs. control group) using Pearson correlation analysis to evaluate module-trait relationships. Modules significantly associated with PC (P < 0.05) were further investigated to identify biologically relevant modules. Genes with high gene significance (GS) values, defined as the absolute correlation between each gene and clinical traits, were considered central genes within important modules. Hub genes were selected using a threshold of |GS| > 0.5 in this study. Finally, a comparative analysis was conducted between the identified DEGs, MMGs, and hub genes obtained from WGCNA. The overlapping genes were considered candidate genes closely associated with mitochondrial metabolism and the development of PC, and were utilized for further functional analysis and validation.

### 2.5 Enrichment analysis of candidate genes

To delve deeper into the biological functions involved in the development of PC, we further conducted functional enrichment analysis on the candidate genes. The clusterProfiler package was utilized for functional enrichment analysis based on Kyoto Encyclopedia of Genes and Genomes (KEGG) pathways and Gene Ontology (GO) categories. The GO annotation includes three categories: Biological Processes (BPs), Cellular Components (CCs), and Molecular Functions (MFs). By calculating the enrichment level of candidate genes in each KEGG pathway or GO term and performing statistical tests using Fisher's exact test, significantly enriched KEGG pathways and GO terms (P < 0.05) were identified. This analysis aimed to reveal the main biological functions and signaling pathways involved in PC related to mitochondrial metabolism.

### 2.6 Selection of key genes using machine learning algorithms and construction of a Nomogram

To further identify the most promising diagnostic gene targets in the classification of PC and normal tissues, we applied five machine learning algorithms - Random Forest (RF), Extreme Gradient Boosting (XGBOOST), Least Absolute Shrinkage and Selection Operator (LASSO), Gradient Boosting Machine (GBM), and Support Vector Machine Recursive Feature Elimination (SVM-RFE) – to the previously identified candidate genes. The “randomForest” package was used for the Random Forest algorithm, which evaluates the importance of each gene by calculating the average reduction in impurity across all decision trees. The “xgboost” package in R software was used for XGBOOST analysis, which efficiently selects important features. The “glmnet” package was employed for LASSO analysis, which compresses coefficients of irrelevant or redundant features to zero using sparse regularization for feature selection. The “gbm” package was utilized for GBM analysis, which identifies important features based on their importance scores in optimizing the target. Lastly, the “e1071” package was used for SVM-RFE algorithm implementation, which iteratively removes the least important features based on the weights assigned by the SVM classifier. Additionally, the MCC algorithm in the Protein-Protein Interaction (PPI) network was employed for candidate gene. The cross genes selected by the five machine learning algorithms combined with the MCC algorithm were considered as key genes for the diagnosis and progression of PC. Subsequently, to accurately assess the risk of PC development, a logistic regression nomogram risk prediction model was developed using the key genes as variables and the PC occurrence rate as the outcome variable. A multivariate logistic regression model was built, assigning a score to each key gene based on the regression coefficients and summing these scores to obtain the total score for constructing the nomogram. The total score was then converted into the corresponding predicted probability of PC occurrence. Calibration curves were used to evaluate the model performance, and decision curve analysis (DCA) was employed to assess the clinical utility of the nomogram by quantifying the net benefit at different probability thresholds.

### 2.7 Immunoinfiltration analysis

This study utilized the CIBERSORT algorithm to analyze gene expression data of PC tissue and control tissue samples, exploring the relative abundance of 22 immune cell subtypes. Differences in immune cell infiltration between the two groups were compared using the Wilcoxon rank-sum test to identify significantly enriched or reduced immune cell types in tumor tissue (P < 0.05). Additionally, Pearson correlation analysis was used to assess the correlation between different immune cell subtypes, revealing interactions and synergistic effects of immune cells in the tumor microenvironment (r > 0.3, P < 0.05). The Pearson correlation coefficient was also calculated between the expression levels of each key gene and the abundance of various immune cell subtypes to investigate the relationship between key genes and immune cell infiltration (r > 0.3, P < 0.05), inferring the potential role of these genes in regulating the tumor immune microenvironment.

### 2.8 Network regulatory mechanism of key genes

To explore the putative regulatory mechanisms of key genes based on computational prediction in the development of PC, this study conducted analyses from three aspects: competitive endogenous RNA (ceRNA) regulation, transcription factor (TF) regulation, and protein-protein interaction (PPI). Firstly, to investigate the ceRNA regulatory relationships between different types of RNA molecules, a ceRNA regulatory network containing lncRNA, miRNA, and mRNA was constructed. Predictions of miRNA interactions with key genes were made using the miRDB database (https://mirdb.org/) and mirDIP database (https://ophid.utoronto.ca/mirDIP/), while LncBase database (https://dianalab.e-ce.uth.gr/html/diana/web/index.php?r=lncbasev2) was used to predict upstream regulatory lncRNAs of miRNA. The predicted miRNA-mRNA and lncRNA-miRNA interaction relationships were utilized to build the ceRNA network. The network was visualized using Cytoscape software to demonstrate the potential multi-level regulatory mechanisms of key genes. Furthermore, to uncover the TFs involved in regulating the expression of key genes, the study conducted TF prediction analysis using the hTFtarget database (https://guolab.wchscu.cn/hTFtarget/#!/tf) to identify important upstream transcriptional regulators of key genes. At the protein level, the study employed the STRING database (https://string-db.org/) for protein-protein interaction (PPI) analysis of candidate genes, selecting interaction pairs with confidence scores ≥ 0.4. Subsequently, a PPI network was constructed using Cytoscape software, and the Maximal Clique Centrality (MCC) algorithm was used to assign importance scores to nodes in the network. Finally, key genes were placed at the network center to visually illustrate their core regulatory role in the PPI network and their potential interactions with other candidate genes.

### 2.9 Drug prediction and molecular docking analysis

This study utilized the DSigDB database (https://maayanlab.cloud/Enrichr/) to predict potential small molecule compounds interacting with key genes. The key genes were uploaded to the platform, and based on the ranking by p-value, the comprehensive score indicated the potential of the corresponding small molecule compounds to interact with the genes.

To validate the reliability of the small molecule drugs predicted by DSigDB and to identify drugs targeting key genes, molecular docking analysis was conducted to assess the interaction strength and mode between the small molecule drugs and key target proteins. The three-dimensional structures of candidate small molecule drugs were downloaded from the PubChem database (https://pubchem.ncbi.nlm.nih.gov/). The AutoDockTools software package was used for pre-processing the small molecules and target proteins, including steps such as hydrogenation, calculating Gasteiger charges, etc. Based on the structural information of the target proteins, the docking region was defined, and docking parameters were set. Molecular docking simulations were performed using AutoDock Vina software to calculate the binding free energy of each small molecule with the target protein and analyze their binding modes, such as hydrogen bonding, hydrophobic interactions, etc. The “ligand-receptor” complexes with the lowest binding energy (strongest binding) for each key target were visually analyzed. The binding site of the small molecule drugs with the target protein, the key groups involved in the interaction, and the molecular interactions formed were visually displayed using the Pymol software.

### 2.10 External validation of key genes and Receiver Operating Characteristic (ROC) curve analysis

To confirm whether the expression trends of key genes in the training and validation sets were consistent, the expression of candidate genes in PC and control samples was analyzed in the training and validation sets (P < 0.05). ROC curve analysis was conducted using the “pROC” package, and genes with AUC > 0.7 in both the training and validation sets were considered to have good discriminatory ability. Additionally, to validate the expression of key genes at the single-cell level, the key genes were regressed into single-cell data for internal validation. The expression levels of key genes in various cell types, their distribution between PC and control groups, and their expression in specific cells were analyzed.

### 2.11 Prognostic value of key genes in PC

To explore the predictive ability of key genes for the prognosis of PC, the optimal cut-off value for gene expression was calculated using the surv_cutpoint function. Tumor samples from PC from TCGA were divided into high- and low- expression groups. Kaplan-Meier (KM) survival analysis was performed using the “survival” package to evaluate the difference in survival between the two groups.

## 3 Results

### 3.1 scRNA-seq single-cell data analysis

**Fig 1:**
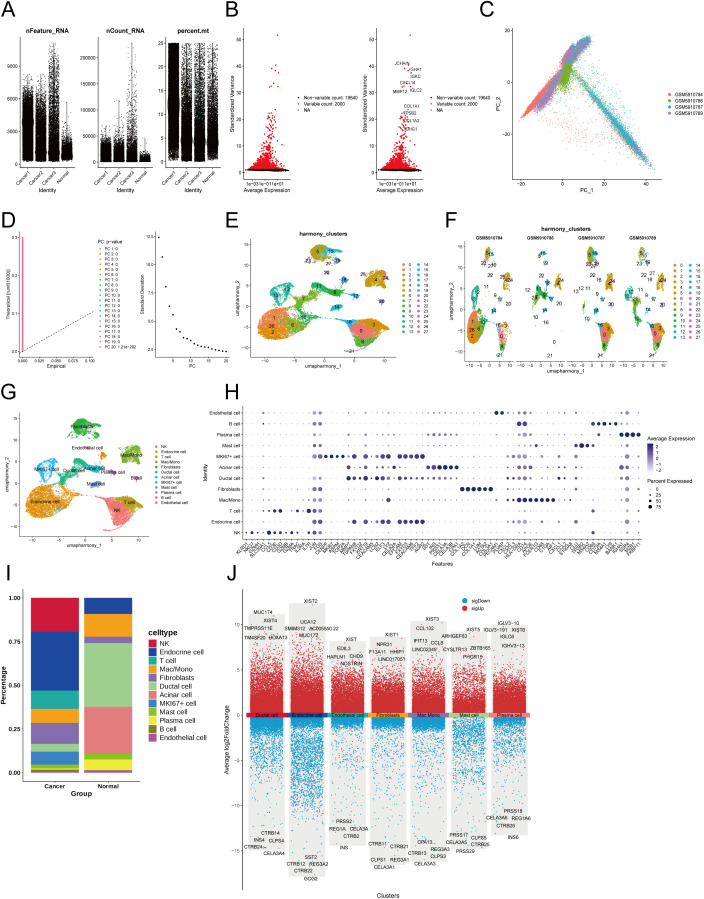
Single-cell Data Analysis Reveals Cellular Heterogeneity and Cell Distribution in Pancreatic Cancer (PC) Tissue. **(A)** Scatter plot of cell quality control for single-cell sequencing samples. **(B)** Selection of highly variable genes. Red dots represent highly variable genes, while black dots represent non-variable genes. **(C-D)** Principal Component Analysis. **(E)** UMAP cell dimensionality reduction clustering. Different colors represent different cell subtypes. **(F)** Classification of cell clusters in each sample. **(G)** UMAP dimensionality reduction clustering cell annotation plot. **(H)** Expression levels of marker genes for each cell cluster. **(I)** Proportional representation of cells between PC and normal groups. **(J)** Volcano plot of differential expression genes for each cell cluster.

Based on the single-cell dataset GSE197177, preprocessing was performed using the “Seurat” package to filter out unqualified cells, extract core cells from the dataset, and normalize the data for subsequent analysis (Fig 1A). Variance analysis was conducted on the core cells in the single-cell dataset to identify genes with strong variability, and the top 10 most variable genes were labeled, such as JCHAIN, IGHA1, IGKC, and CXCL14, among others (Fig 1B). Through Principal Component Analysis (PCA), the top 20 PCs were determined based on statistical significance (P < 0.05) and explained variance, which collectively captured the major sources of variation in the dataset (Fig 1C, 1D). Subsequently, the UMAP algorithm was utilized to classify the core units into 28 independent cell clusters (Fig 1E, 1F). It should be emphasized that the quality control, PCA, and clustering panels presented in Fig 1A–1F serve as the methodological foundation of the scRNA-seq analysis pipeline rather than the principal findings of this study.

Cell annotations were performed for different clusters, and the expression of important marker genes for each cell type was visualized using bubble plots. The annotation revealed 12 cell clusters, including NK cells, T cells, MKI67 + cells, ductal cells, endocrine cells, acinar cells, macrophage (Mac)/monocyte (Mono) cells, mast cells, B cells, plasma cells, fibroblasts, and endothelial cells (Fig 1G, 1H). Among them, endothelial cells were the most abundant cells in the PC microenvironment, while NK cells, T cells, and fibroblasts were also major components of the tumor microenvironment (Fig 1I). Finally, differential expression genes among different cell types were analyzed using the FindMarkers function with statistical thresholds of adjusted P-value < 0.05 and |log2FC| > 0.5, and these genes were visualized (Fig 1J). Given the established role of fibroblasts in mitochondrial metabolism regulation in PC as reported in previous studies [24], we specifically focused our analysis on this cell population. This analysis identified 8,231 scRNA-DEGs in fibroblasts (adjusted P-value < 0.05, |log_2_FC| > 0.5), including 6,539 up-regulated genes and 1,692 down-regulated genes.

### 3.2 Differential expression analysis of transcriptome data

**Fig 2:**
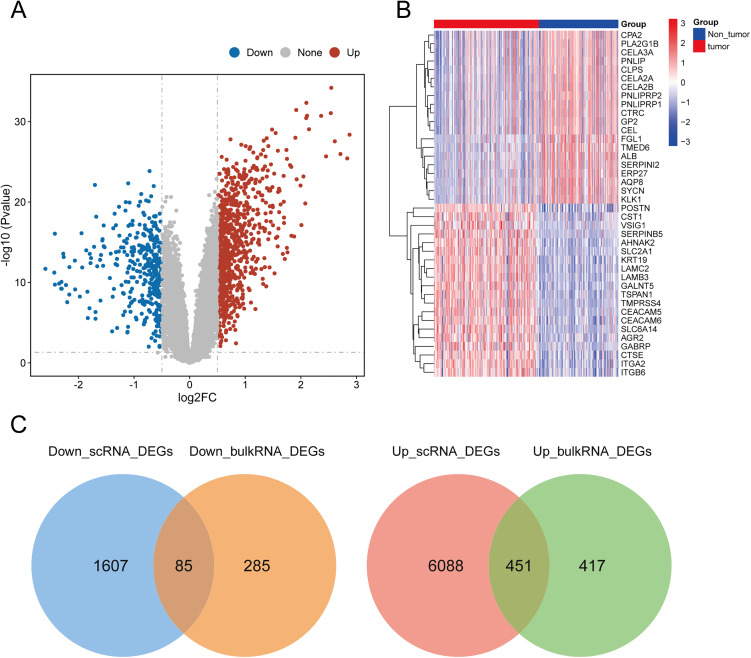
Differential Expression Analysis and Acquisition of Differential Expression Genes (DEGs). **(A)** Volcano plot of bulk RNA-DEGs. **(B)** Heatmap of the top 20 up-regulated and down-regulated bulk RNA-DEGs. **(C)** Venn diagram showing the intersection of scRNA-DEGs and bulk RNA-DEGs.

Differential expression analysis was performed on the transcriptome data of the training set using appropriate statistical methods, resulting in the identification of bulk RNA-DEGs. With the criteria of |log_2_FC| > 0.5 and P < 0.05, a total of 1,238 bulk RNA-DEGs were found to be significantly differentially expressed in the tumor group compared to the normal samples in PC, including 868 up-regulated genes and 370 down-regulated genes (Fig 2A, 2B). Subsequently, to identify robust DEGs that are consistently dysregulated at both single-cell and bulk tissue levels, we took the intersection of scRNA-DEGs and bulk RNA-DEGs. This cross-validation approach helps to reduce false positives and identifies genes with reproducible expression changes across different analytical platforms and cellular contexts. The intersection analysis resulted in 536 DEGs, which comprised 85 down-regulated genes and 451 up-regulated genes (Fig 2C). These overlapping genes represent a high-confidence set of dysregulated genes that are detectable in both the cellular resolution of single-cell analysis and the tissue-level bulk RNA sequencing, making them more reliable candidates for downstream functional analysis.

### 3.3 WGCNA

**Fig 3:**
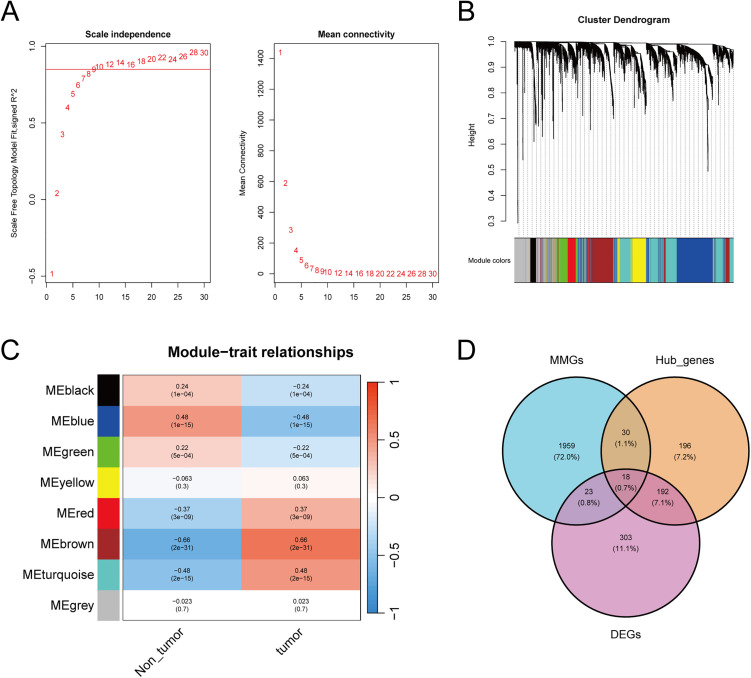
WGCNA. **(A)** Data set queue independence and soft thresholding, as well as connectivity. **(B)** Cluster dendrogram. **(C)** Heatmap of the correlation analysis between module characteristic genes and disease phenotypes. **(D)** Venn diagram showing the acquisition of candidate genes.

The gene expression profile data was utilized for WGCNA to identify module genes related to PC. The pickSoftThreshold function was used to test different soft-thresholding powers (β) and construct an adjacency matrix based on the selected optimal soft-threshold (β = 9) (Fig 3A), which was further transformed into a Topological Overlap Matrix (TOM) to reflect the co-expression similarity between genes. Subsequently, based on hierarchical clustering and dynamic tree cut algorithm, with a minimum module gene size set to 30 and a cut height set to 0.25, a total of 8 gene modules were generated (Fig 3B). Module Eigengenes (MEs) were calculated and correlated with clinical traits (tumor vs. control) using Pearson correlation analysis to assess module-trait relationships. The results showed that 6 modules (black, blue, green, brown, turquoise, and red) were significantly correlated with the disease phenotypes (P < 0.05) (Fig 3C), indicating that these modules may be closely associated with the occurrence and development of PC. From the significantly correlated modules, hub genes were identified based on gene significance (GS) threshold of |GS| > 0.5, resulting in 436 hub genes with high correlation to PC clinical traits. To identify key genes that are both differentially expressed in PC and functionally involved in mitochondrial metabolism regulation, we performed a three-way intersection analysis combining: (1) 436 hub genes from WGCNA analysis, which represent genes with central roles in disease-associated co-expression networks based on their strong correlation with PC phenotype; (2) 536 DEGs identified from the intersection of single-cell and bulk RNA-seq analyses (|log_2_FC| > 0.5, P < 0.05), which represent consistently dysregulated genes across different analytical platforms; and (3) mitochondrial metabolism-related genes (MMGs), which represent genes with established roles in mitochondrial function. This integrated multi-omics approach ensures that candidate genes are not only statistically significant but also biologically relevant to both PC pathogenesis and mitochondrial metabolic dysregulation. The three-way intersection resulted in 18 candidate genes (Fig 3D). These 18 genes were not only significantly differentially expressed in PC but also closely related to the regulation of mitochondrial metabolism, making them high-confidence candidates for subsequent functional analysis and validation.

### 3.4 Enrichment analysis of candidate genes

To elucidate the biological functions and pathways associated with the 18 candidate genes, GO and KEGG enrichment analyses were performed using the clusterProfiler package. Fisher's exact test was employed to assess the statistical significance of enrichment, with a threshold of P < 0.05 to identify significantly enriched terms and pathways. The top 5 significantly enriched GO terms in each category (Biological Processes, Cellular Components, and Molecular Functions) and top 10 KEGG pathways were visualized based on gene count and P-values.

**Fig 4:**
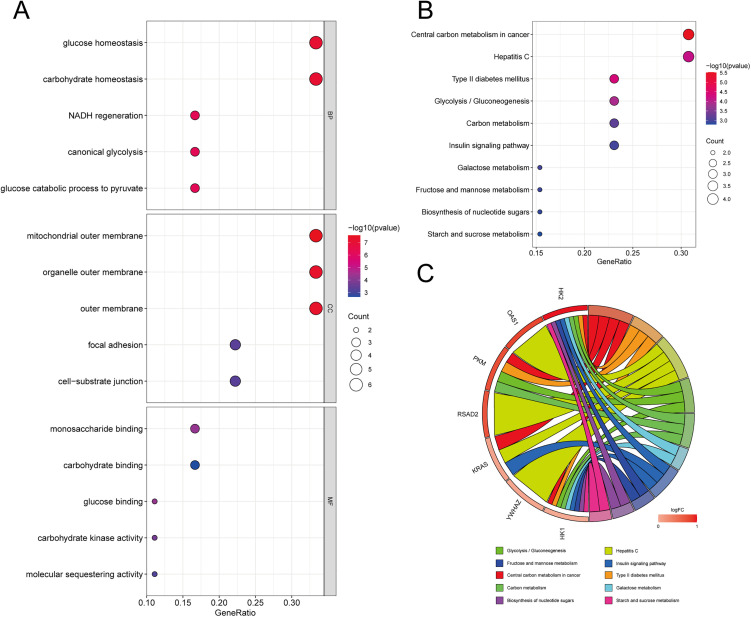
GO and KEGG Enrichment Analysis. **(A)** GO enrichment analysis. **(B)** KEGG enrichment analysis. **(C)** Connection between candidate genes and enriched KEGG pathways.

In the GO enrichment analysis, these genes were predominantly enriched in biological processes such as glucose homeostasis, carbohydrate homeostasis, NADH regeneration, canonical glycolysis, and glucose catabolic process to pyruvate, highlighting their critical roles in energy metabolism reprogramming characteristic of pancreatic cancer cells; cellular components including mitochondrial outer membrane, organelle outer membrane, outer membrane, focal adhesion, and cell-substrate junction, indicating their dual involvement in both mitochondrial structural organization and cellular adhesion machinery; and molecular functions such as glucose binding, monosaccharide binding, carbohydrate kinase activity, molecular sequestering activity, and carbohydrate binding, suggesting their regulatory roles in glucose sensing and metabolism (Fig 4A).

The KEGG enrichment analysis revealed that these genes were significantly involved in pathways such as central carbon metabolism in cancer, type II diabetes mellitus, hepatitis C, glycolysis/gluconeogenesis, and carbon metabolism (Fig 4B, 4C). These enrichment patterns provide several important biological insights. First, the prominent enrichment in glycolysis and central carbon metabolism pathways strongly supports the metabolic reprogramming hypothesis in PC, where tumor cells exhibit enhanced glycolytic activity (Warburg effect) to meet their increased biosynthetic and energetic demands. Second, the significant enrichment of mitochondrial outer membrane-related terms confirms that these candidate genes are indeed closely associated with mitochondrial structure and function, validating our initial hypothesis linking mitochondrial metabolism to PC pathogenesis. Third, the enrichment in focal adhesion and cell-substrate junction suggests that these mitochondrial metabolism-related genes may also regulate tumor cell migration and invasion through cytoskeletal remodeling, highlighting the interconnection between metabolic alterations and metastatic potential in PC. Fourth, the association with type II diabetes mellitus pathway is clinically relevant, as epidemiological studies have established diabetes as both a risk factor and consequence of pancreatic cancer, suggesting shared molecular mechanisms. Collectively, these functional enrichment results demonstrate that the 18 candidate genes play multifaceted roles in PC by orchestrating metabolic reprogramming, maintaining mitochondrial homeostasis, and potentially influencing tumor invasiveness, thereby providing important mechanistic insights and identifying potential therapeutic targets for metabolic intervention in PC treatment.

### 3.5 Machine learning for key gene selection

**Fig 5:**
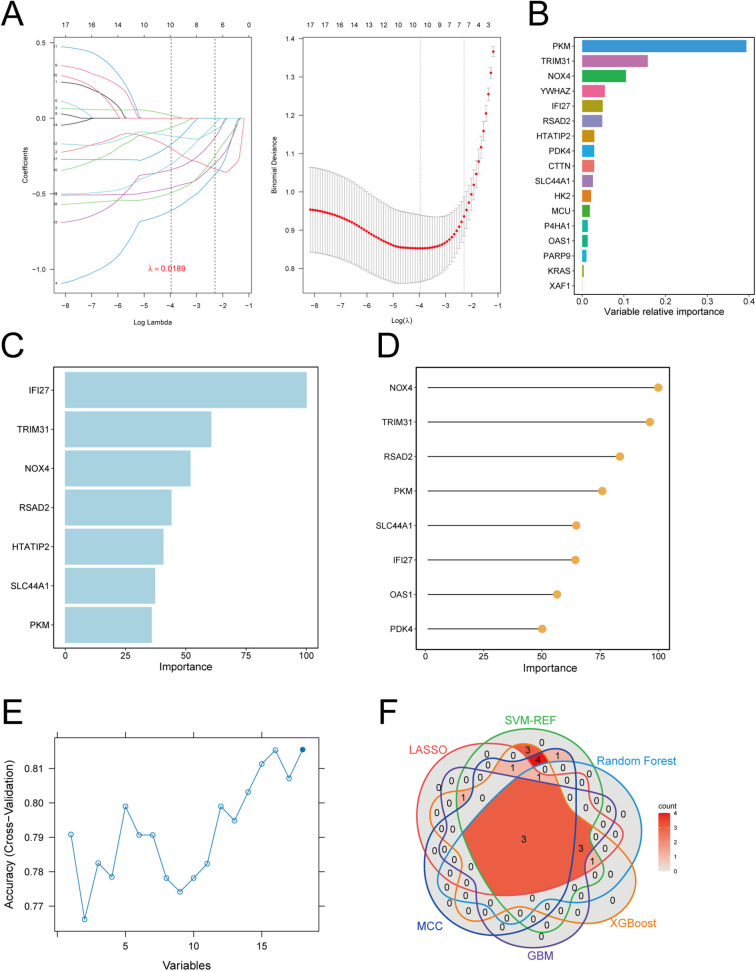
Machine Learning Algorithms and PPI Selecting Key Genes. **(A)** LASSO model. **(B)** XGBoost algorithm. **(C)** GBM algorithm. **(D)** Random Forest for significant gene selection. **(E)** SVM-RFE for significant gene selection. **(F)** Venn diagram showing the intersection of key genes selected by five machine learning algorithms and PPI.

Five different machine learning algorithms (XGBoost, SVM-RFE and Random Forest, LASSO, GBM) were applied to select 18 candidate genes. Firstly, the LASSO algorithm was used for gene selection, resulting in 10 feature genes: PKM, PDK4, SLC44A1, IFI27, HK2, OAS1, HTATIP2, NOX4, RSAD2, TRIM31 (Fig 5A). Next, gene selection was performed using XGBoost analysis, leading to the identification of 17 feature genes: PKM, TRIM31, NOX4, YWHAZ, IFI27, RSAD2, HTATIP2, PDK4, CTTN, SLC44A1, HK2, MCU, P4HA1, OAS1, PARP9, KRAS, XAF1 (Fig 5B). The GBM algorithm screened 7 feature genes: PKM, SLC44A1, IFI27, HTATIP2, NOX4, RSAD2, TRIM31 (Fig 5C). Random Forest algorithm was employed for gene selection, yielding 8 feature genes: NOX4, TRIM31, RSAD2, PKM, SLC44A1, IFI27, OAS1, and PDK4 (Fig 5D). Subsequently, 18 feature genes were figured out using the SVM-RFE algorithm, namely PKM, SLC44A1, NOX4, IFI27, CTTN, OAS1, HTATIP2, HK2, XAF1, YWHAZ, RSAD2, P4HA1, TRIM31, MCU, HK1, PDK4, PARP9, KRAS (Fig 5E). Finally, the genes selected by the five algorithms were intersected with the 10 genes obtained from the PPI screening (IFI27, RSAD2, OAS1, XAF1, PARP9, PKM, KRAS, HK1, HK2, YWHAZ), and 3 key genes were obtained, including IFI27, PKM, and RSAD2 (Fig 5F).

### 3.6 Immune infiltration analysis

**Fig 6:**
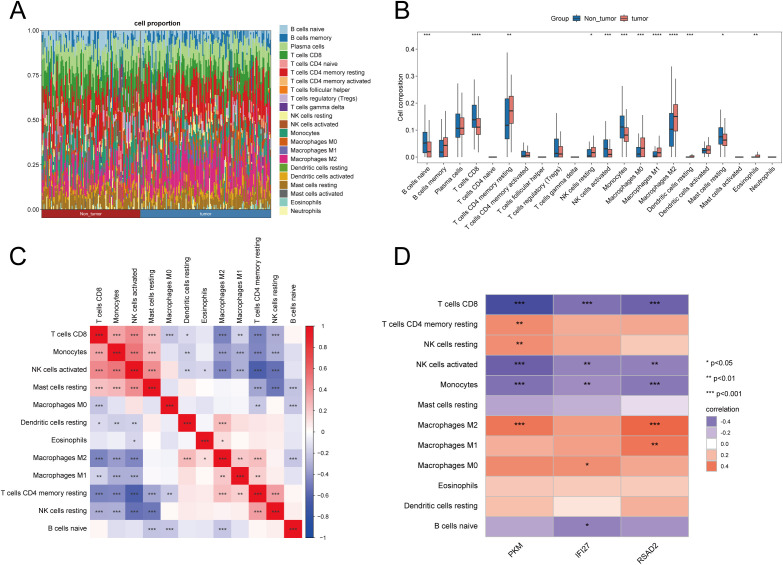
Immune Infiltration Analysis. **(A)** Proportions of immune cell infiltration in control (non-tumor) and PC (tumor) group samples. **(B)** Analysis of differences in immune cell infiltration proportions between control (non-tumor) and PC (tumor) group samples. **(C)** Heatmap analysis of correlations between different immune cell subtypes. **(D)** Heatmap showing the correlations between key genes and different immune cell subtypes.

To further explore the immune microenvironment of PC, the CIBERSORT algorithm was used in the training set to study the infiltration of immune cells in the control (non-tumor) and PC (tumor) samples. A total of 22 types of immune cell subtypes were identified in two groups, and the stacked bar plot shows the types and quantities of immune cell infiltration in each sample (Fig 6A). Next, we compared the differences in infiltration of each immune cell subtype between the non-tumor and tumor groups. The results showed that seven immune cell subtypes, including T cells CD4 memory resting, NK cells resting, macrophages M0, macrophages M1, macrophages M2, dendritic cells resting, and eosinophils, were significantly up-regulated in PC samples (P < 0.05). Conversely, immune cell subtypes such as B cells naive, T cells CD8, NK cells activated, monocytes, and mast cells resting were significantly down-regulated in PC samples (P < 0.05) (Fig 6B). Furthermore, we assessed the correlation between these immune cell populations and found that NK cells activated exhibited the strongest negative correlation with T cells CD4 memory resting, while NK cells activated showed the strongest positive correlation with monocytes (Fig 6C). Additionally, we further studied the correlation between the expression of three key genes and the infiltration of each immune cell subtype. The results showed that the key gene IFI27 was significantly positively correlated with macrophages M0 (r > 0.3, P < 0.05) and significantly negatively correlated with T cells CD8, NK cells activated, monocytes, and B cells naive (r < −0.3, P < 0.05) (Fig 6D). PKM was significantly positively correlated with T cells CD4 memory resting, NK cells resting, and macrophages M2, and significantly negatively correlated with T cells CD8, NK cells activated, and monocytes. RSAD2 was significantly positively correlated with macrophages M1 and macrophages M2, and significantly negatively correlated with T cells CD8, NK cells activated, and monocytes. These results further validate that key genes related to mitochondrial metabolism may play a crucial role in the occurrence and development of PC by regulating the infiltration of specific immune cell subtypes mentioned above.

### 3.7 Construction and evaluation of Nomogram

**Fig 7:**
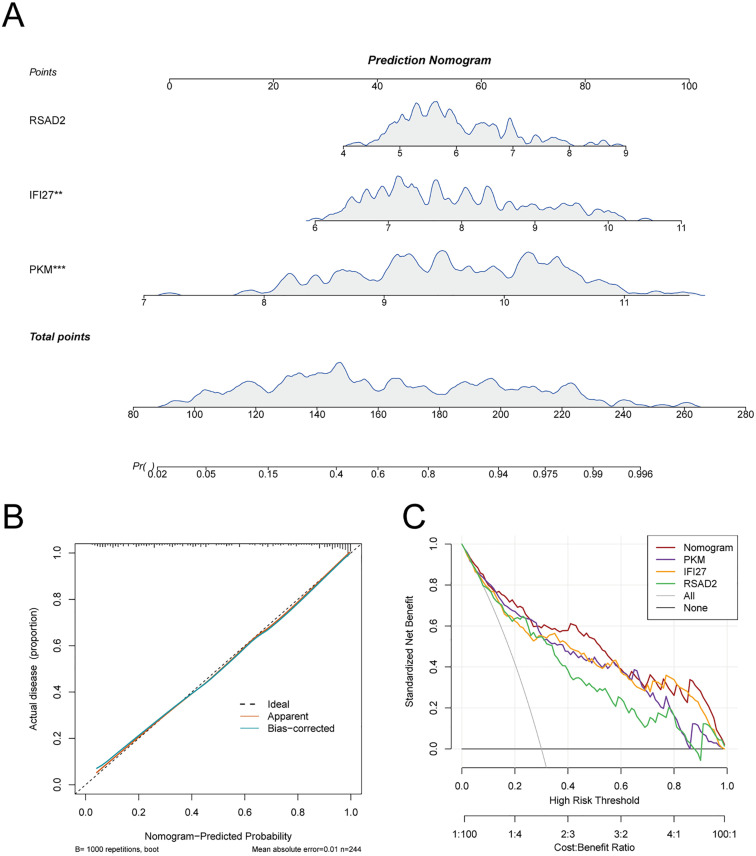
Risk Prediction Model and Evaluation Based on Key Genes. **(A)** Construction of the nomogram risk prediction model. **(B)** Calibration curve. **(C)** Decision curve of the model.

Based on 3 key genes, a nomogram risk prediction model was constructed to assess the risk of PC. The results indicated that as the expression of the three genes increased, the risk factors for PC also increased (Fig 7A). The calibration curve showed good consistency between predicted probability and actual occurrence probability, indicating high predictive accuracy of the model (Fig 7B). Results from DCA demonstrated that within a relatively wide range of threshold probabilities, the net benefits of using the nomogram for prediction were superior to the two extreme scenarios of predicting all patients or no patients, confirming the potential clinical utility of the nomogram risk prediction model (Fig 7C). In conclusion, the nomogram risk prediction model based on the 3 key genes exhibited good predictive ability and accuracy.

### 3.8 Network regulatory mechanism of key genes

**Fig 8:**
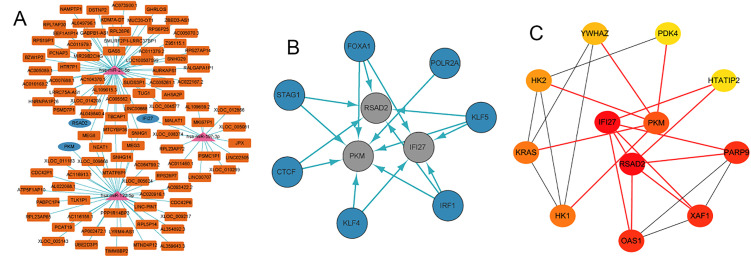
Network Regulatory Mechanism of Key Genes. **(A)** The lncRNA-miRNA-key gene regulatory network. Orange nodes represent lncRNAs, pink nodes represent miRNAs, and blue nodes represent mRNAs. **(B)** TF-mRNA regulatory network. Blue nodes represent TFs, and gray nodes represent key genes. **(C)** PPI network. The color depth of nodes is related to the centrality degree (Degree value), with darker colors indicating higher centrality degree.

To explore putative regulatory mechanisms of key genes based on computational prediction in PC, this study conducted analyses from three aspects: ceRNA regulation, TF regulation, and PPI. At the post-transcriptional level of key genes, the ceRNA network consists of 3 key mRNAs, 3 miRNAs, 97 lncRNAs and 105 interactions (Fig 8A). Multiple lncRNAs can competitively bind to the same miRNA, thereby influencing the expression levels of downstream key genes. For example, lncRNAs SNHG1 and NEAT1 may indirectly regulate the expression levels of PKM and RSAD2 by competitively binding to hsa-miR-122-5p and hsa-miR-21-5p. These results suggest that the interaction between lncRNAs and miRNAs may be one of the important mechanisms regulating the expression of key genes in PC.

According to the prediction from the hTFtarget database, 7 potential TF binding sites were significantly enriched in the promoter regions of the key genes, including FOXA1, POLR2A, KLF5, IRF1, KLF4 and so on. Among them, FOXA1, IRF1 and KLF5 were shared TFs for the three key genes, indicating their potential roles in co-regulating the expression of key genes and promoting PC progression (Fig 8B).

Furthermore, the PPI analysis revealed a network containing 12 nodes and 23 edges, indicating strong interactions among the candidate genes and potential synergistic effects in PC progression. By evaluating the importance of each node in the PPI network using the MCC algorithm, it was found that the key genes IFI27, RSAD2, and PKM are predicted to have close interactions in the PPI network. They had direct or indirect interactions with genes such as PARP9, XAF1, OAS1, HK1 and KRAS (Fig 8C).

### 3.9 Drug prediction and molecular docking

**Fig 9:**
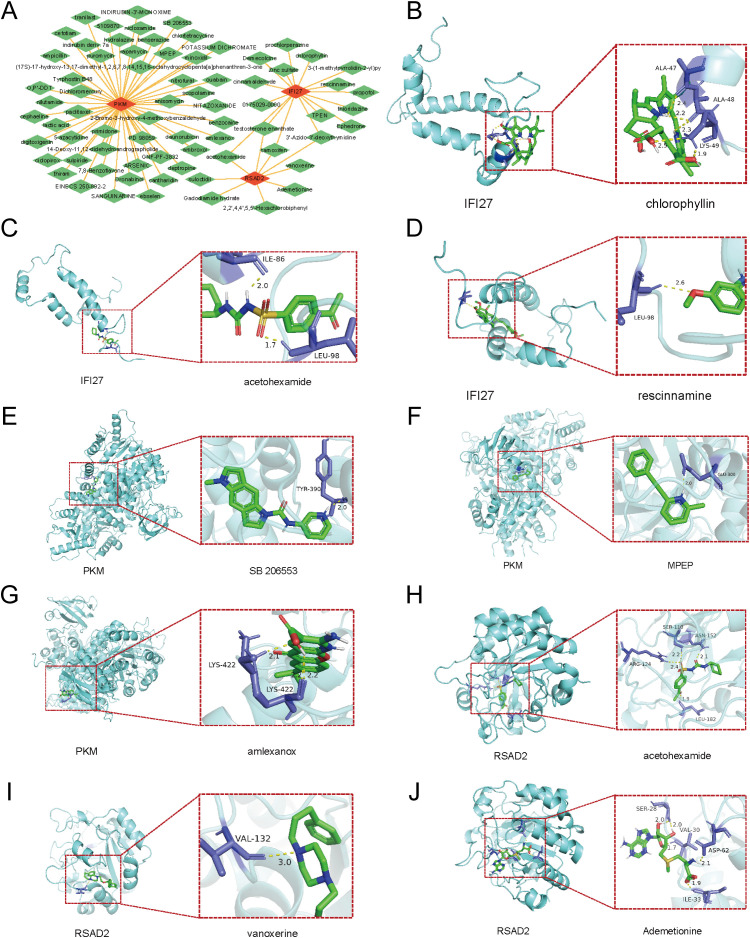
Drug Prediction and Molecular Docking Visualization. **(A)** Key gene-drug prediction network. **(B-J)** Interaction modes between key genes and targeted drugs.

The key genes in PC were uploaded to the DSIGDB database, resulting in the identification of 76 small-molecule drugs with the most potential to improve PC (P < 0.05). These drugs included acetohexamide, ademetionine, vanoxerine, amlexanox, SB 206553, MPEP, rescinnamine, and chlorophyllin (Fig 9A). Additionally, molecular docking experiments were conducted to analyze the strength and mode of interaction between small-molecule drugs and key targets. The results (Table 1) showed that the ligand acetohexamide had the strongest targeted binding with the receptor RSAD2, acetohexamide had the strongest binding with IFI27, and amlexanox had the strongest targeted binding with PKM. Furthermore, visualizations of the “ligand-receptor” complexes for each key target were performed to intuitively demonstrate the interaction modes between small-molecule drugs and target proteins (Fig 9B–9J).

**Table 1:** Binding free energies between key genes and small molecule drugs (kcal/mol).

Receptor	Ligand	Combined score	P-value	Binding energy
IFI27	acetohexamide	135204.64	1.15E-03	−6.86
IFI27	rescinnamine	134347.69	1.20E-03	−6.84
IFI27	chlorophyllin	129851.14	1.50E-03	−5.99
PKM	amlexanox	146675.88	6.50E-04	−8.45
PKM	SB 206553	145187.41	7.00E-04	−7.58
PKM	MPEP	142504.37	8.00E-04	−6.29
RSAD2	acetohexamide	135204.64	1.15E-03	−9.17
RSAD2	Ademetionine	115169.25	3.10E-03	−5.45
RSAD2	vanoxerine	110765.84	3.85E-03	−7.23

### 3.10 External validation and ROC curve analysis

**Fig 10:**
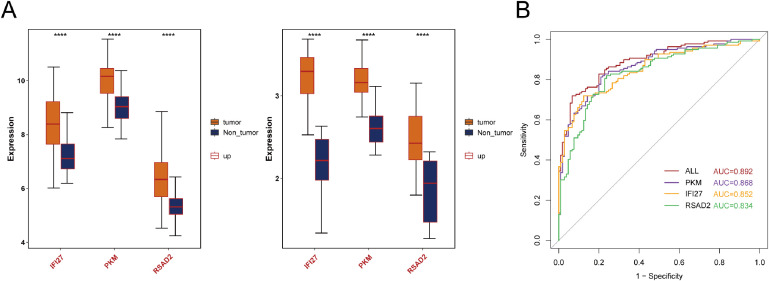
External Validation and ROC Analysis of Key Genes. **(A)** Analysis of the expression levels of key genes (IFI27, PKM, and RSAD2) in the training set (left) and validation set (right). **(B)** ROC curve analysis.

To evaluate the expression patterns of key genes and their diagnostic performance in PC, Wilcoxon rank-sum tests were used to analyze the expression levels of these key genes in the training and validation sets. Compared to the normal control group (non-tumor), all three key genes were significantly up-regulated in the PC (tumor) group (P < 0.05), and the expression trends of key genes were consistent in both the training and validation sets (Fig 10A), indicating their potential as risk factors for PC. ROC curve analysis further showed that the AUC values of the 3 key genes (IFI27, PKM, and RSAD2) were 0.852, 0.868, and 0.834, respectively. The combined diagnostic prediction AUC for the three genes was 0.892, demonstrating their good diagnostic capabilities (Fig 10B).

**Fig 11:**
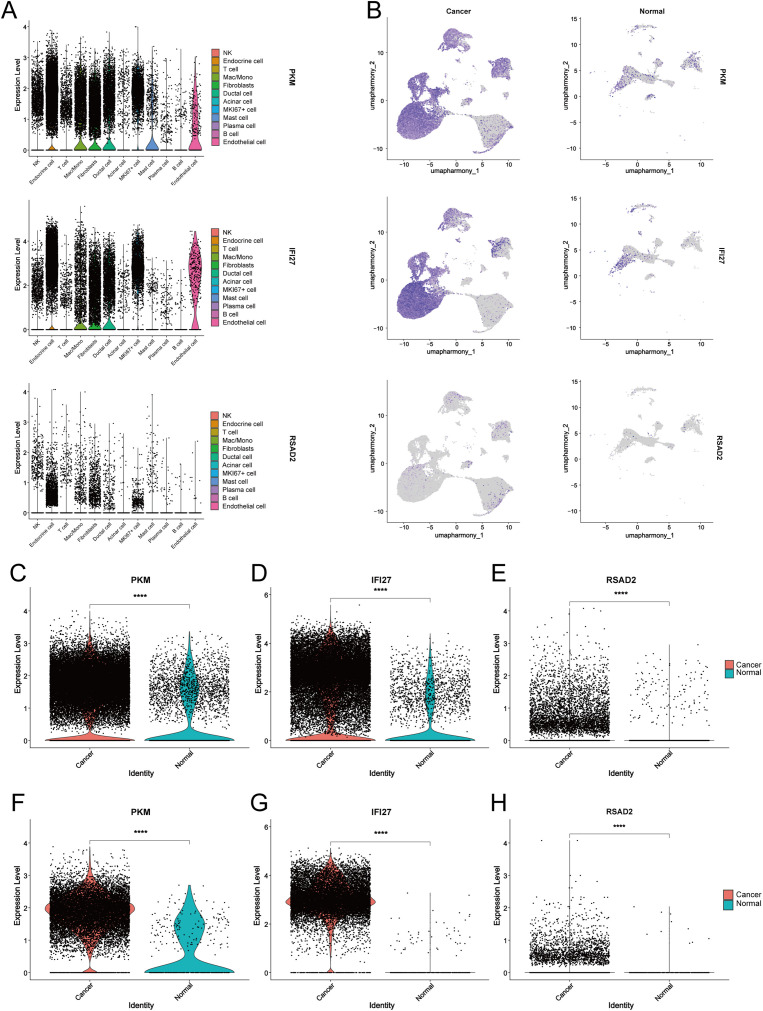
Expression Validation of Key Genes at the Single-Cell Level. **(A)** Expression of key genes in various cell subtypes. **(B)** Spatial distribution of key genes in PC and control samples. **(C-E)** Quantitative expression levels of PKM **(C)**, IFI27 **(D)**, and RSAD2 (E) in PC and control groups (**** represents P < 0.0001). **(F-G)** Quantitative expression levels of PKM **(C)**, IFI27 **(D)**, and RSAD2 (E) in endocrine cells of PC and control groups (**** represents P < 0.0001).

Finally, internal validation was conducted by examining the expression of key genes in single-cell data. The results showed that these key genes were expressed in various cell clusters, with PKM and IFI27 being expressed in fibroblasts, Mac/Mono, MKI67 + cells, ductal cells, and endocrine cell, while RSAD2 was expressed in endocrine cell, fibroblasts, Mac/Mono, MKI67 + cells, and NK cells. Overall, PKM, IFI27, and RSAD2 were mainly expressed in endocrine cells (Fig 11A). Subsequent analysis at the single-cell level revealed differences in the expression distribution of key genes between PC and control sample groups. UMAP visualization results showed that the expression of these three genes in PC samples was significantly higher than in the normal control group (Fig 11B). Quantitative analysis further confirmed that the expression of the three genes was significantly up-regulated in the PC group (Fig 11C–11E). Furthermore, to explore the expression differences of key genes in specific cell subtypes of PC and control groups, we extracted the endocrine cell subtype and analyzed the expression differences of key genes. It was found that the expression levels of these three key genes were significantly up-regulated in the endocrine cells of the PC group (Fig 11F–11H). These results suggest that PKM, IFI27, and RSAD2 may play important roles in the occurrence and development of PC, with potential diagnostic and therapeutic value.

### 3.11 Survival analysis

**Fig 12:**
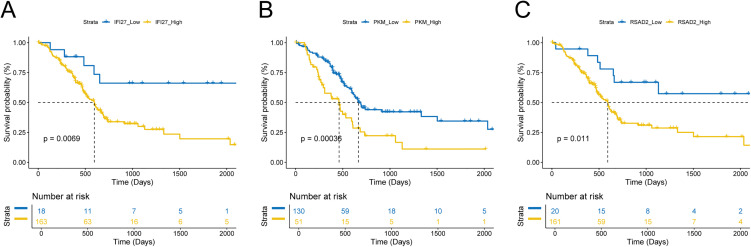
Survival Analysis of Key Genes. **(A)** Kaplan-Meier survival curves predicting PC survival outcomes for high and low expression groups of IFI27 in TCGA. **(B)** Kaplan-Meier survival curves predicting PC survival outcomes for high and low expression groups of PKM in TCGA. **(C)** Kaplan-Meier survival curves predicting PC survival outcomes for high and low expression groups of RSAD2 in TCGA.

We investigated the association between the expression of key genes in PC and their prognostic value. The relationship between PC survival outcomes and the expression of key genes was evaluated using Kaplan-Meier survival curves from the TCGA database. The results showed that compared to low expression, the high expression groups of the three key genes exhibited poorer prognostic survival outcomes (P < 0.05) (Fig 12A–12C). These findings suggest that IFI27, PKM, and RSAD2 can serve as effective independent biomarkers for predicting the prognosis of PC.

## 4 Discussion

PC is a highly lethal malignant tumor of the digestive system [25]. Despite advances in treatment, patient prognosis remains poor [3]. Increasing evidence suggests that metabolic reprogramming of the tumor microenvironment plays a crucial role in the development of PC [26]. Among these, fibroblasts, as the main component of the PC microenvironment, provide nutritional support to tumor cells through metabolic reprogramming [27]. However, due to the high heterogeneity of PC tissue, the molecular mechanisms regulating mitochondrial metabolism in different cell subpopulations have not been fully elucidated. Therefore, a deeper investigation into the mitochondrial metabolic characteristics and regulatory mechanisms of specific cell types in PC is essential for understanding tumor progression and developing new therapeutic strategies.

We conducted enrichment analysis on the candidate genes, and the results showed that these genes were mainly enriched in pathways such as Type II diabetes mellitus, glycolysis/gluconeogenesis, and carbon metabolism. These pathways are suggested to represent critical metabolic vulnerabilities in PC. The glycolysis/gluconeogenesis pathway is fundamentally altered in PC, where enhanced glycolysis not only provides material and energy for rapid tumor proliferation but also generates lactate that acidifies the microenvironment, suppressing immune surveillance [28,29]. Critically, metabolic intermediates like acetyl-CoA and α-ketoglutarate function beyond energy carriers—they serve as epigenetic regulators that modulate histone modifications and DNA methylation, thereby affecting oncogenic gene expression [30–32]. The Type II diabetes-PC relationship is bidirectional: hyperinsulinemia and chronic inflammation in diabetic patients promote PC through IGF-1/PI3K/AKT signaling activation [33,34]. Mechanistically, targeting these interconnected pathways could disrupt this metabolic crosstalk, though the metabolic plasticity of PC cells may limit monotherapy efficacy and necessitate combination strategies.

Through a series of bioinformatics analyses, three key genes were screened and identified. However, their functional roles extend beyond biomarkers and require mechanistic interpretation. Interferon Alpha Inducible Protein 27 (IFI27) is an interferon-stimulated gene that plays a crucial role in innate immune responses and tumor development. This study found that IFI27 is significantly upregulated in PC tissues and is associated with poor prognosis. This finding aligns with previous research, which reported high expression of IFI27 in PC and its promotion of tumor cell proliferation and invasion [35]. The paradox of an immune-related gene predicting worse outcomes suggests IFI27 may facilitate immune evasion through chronic interferon signaling that induces T cell exhaustion and PD-L1 upregulation. Additionally, IFI27 has been reported to localize to mitochondria where it disrupts oxidative phosphorylation, our data are consistent with this possibility and conferring chemoresistance. This dual functionality—immunosuppression and metabolic reprogramming—suggests IFI27 as a candidate therapeutic target combining metabolic normalization with immunotherapy [35]. Pyruvate Kinase M1/2 (PKM) is a key rate-limiting enzyme in glycolysis, catalyzing the conversion of phosphoenolpyruvate (PEP) to pyruvate, playing a central role in tumor metabolic reprogramming [36]. Studies have shown that the PKM2 isoform is highly expressed in PC, promoting tumor growth and metastasis through activation of the mTORC1 signaling pathway [37]. These findings are consistent with our results, indicating elevated PKM expression in PC tissues, suggesting its potential involvement in metabolic adaptive changes and its significant role in PC development. Beyond glycolysis, nuclear PKM2 functions as a protein kinase phosphorylating STAT3 and histone H3, directly activating oncogenic transcription. PKM2 exists in equilibrium between inactive dimers and active tetramers—tumor cells maintain high dimer ratios to divert glucose into biosynthetic pathways for nucleotide synthesis and NADPH production [38]. This explains why simple PKM2 inhibition often fails clinically, as cells compensate through alternative metabolic routes. Successful targeting may require PKM2 activators that force tetramer formation, collapsing biosynthetic flux in combination with antioxidant depletion strategies. Radical S-Adenosyl Methionine Domain Containing 2 (RSAD2), also known as Viperin, is an antiviral protein that plays a critical role in innate immune defense [39]. Research indicates high expression of RSAD2 in cancer, accelerating tumor progression [40]. This finding is consistent with our results, showing significant upregulation of RSAD2 in PC and its association with poor prognosis. The oncogenic role of this antiviral protein appears contradictory but can be explained mechanistically: RSAD2 disrupts mitochondrial tRNA synthesis, triggering HIF-1α stabilization that drives pro-metastatic programs including EMT and angiogenesis. In summary, IFI27, PKM, and RSAD2 should be conceptualized as interconnected nodes in a network where metabolic reprogramming, immune evasion, and oncogenic signaling converge, requiring multi-targeted rather than single-agent interventions.

In the tumor microenvironment, infiltrating immune cells play a crucial role in the development of PC. This study found that the expression levels of three key genes—IFI27, PKM, and RSAD2—are closely related to the infiltration of various immune cell subpopulations. Specifically, we discovered that the expression levels of key genes are positively correlated with the infiltration of M0 and M2 macrophages. M2 macrophages have immunosuppressive effects and can inhibit effector T-cell activation by secreting cytokines such as IL-10 and TGF-β, thereby promoting tumor immune escape [41]. Additionally, M0 macrophages represent unprimed macrophages with high plasticity. Under the influence of the tumor microenvironment, M0 macrophages can polarize into M2 macrophages, producing immunosuppressive effects [42]. Our data suggest that IFI27 and RSAD2 actively recruit M0 macrophages through chemokine signaling and promote their M2 polarization via lactate-induced HIF-1α activation. M2 macrophages produce arginase-1 that depletes arginine, starving T cells and causing cell cycle arrest [43]. Furthermore, M2-derived exosomal miRNAs suppress effector T cell functions, amplifying immunosuppression [44]. Therefore, IFI27/RSAD2 inhibition may reprogram the macrophage compartment and could synergize with CSF1R inhibitors. Conversely, we found that the expression levels of IFI27, PKM, and RSAD2 are negatively correlated with the infiltration of anti-tumor effector cells such as CD8 + T cells and activated NK cells. Clinical studies have shown that the infiltration levels of CD8 + T cells and NK cells in PC tissues are positively correlated with patient prognosis [45,46]. Therefore, these key genes may weaken the anti-tumor immune response by inhibiting the infiltration of CD8 + T cells and NK cells, promoting PC immune escape. Furthermore, PKM is also positively correlated with the infiltration of CD4 memory resting T cells. CD4 + memory T cells play dual roles in the anti-tumor immune response of the body; on one hand, they promote the activation and proliferation of effector T cells by secreting cytokines such as IL-2; on the other hand, regulatory CD4 + T cell subsets can inhibit the function of T cells and NK cells by secreting IL-10 and TGF-β [47]. In this study, PKM's positive correlation with the infiltration of CD4 + resting memory T cells suggests that PKM may primarily promote the infiltration of regulatory CD4 + T cell subsets, leading to the formation of an immunosuppressive microenvironment in PC. In summary, IFI27, PKM, and RSAD2 may remodel the PC immune microenvironment through multiple mechanisms, such as regulating macrophage polarization and inhibiting the infiltration of effector T cells and NK cells, thereby accelerating tumor cell escape from immune surveillance and disease progression. This provides important clues for developing new immunotherapy strategies targeting key genes for PC.

Given the critical role of mitochondrial metabolic reprogramming in the development of PC, the development of drugs targeting mitochondrial metabolism has become a research hotspot in the field of cancer treatment. This study conducted drug prediction and molecular docking analysis and found that small molecule compounds such as acetohexamide, amlexanox, and ademetionine may have the potential to improve the prognosis of PC. Acetohexamide is a traditional oral hypoglycemic drug, belonging to the sulfonylurea class. However, studies have shown that sulfonylurea drugs and insulin may increase the incidence of PC in diabetic patients [48]. Despite molecular docking suggesting acetohexamide binds IFI27, the concerning epidemiological association with increased PC risk cannot be ignored. Chronic sulfonylurea use may stimulate β-cell stress and proliferation, increasing mutation risk. Therefore, acetohexamide should not be advanced without validation that it inhibits IFI27 at concentrations below those causing insulin secretion, and without demonstrating that short-term use avoids risks associated with chronic diabetic therapy. Alternative strategies include developing derivatives retaining IFI27 binding but lacking sulfonylurea receptor activity. Amlexanox has been reported to block autophagic flux and enhance the sensitivity of cancer cells to chemotherapeutic drugs by inhibiting TBK1 kinase activity [49]. Additionally, ademetionine, as a methyl donor, can affect the expression of tumor-related genes by regulating DNA methylation and histone modification [50]. The molecular docking results of this study further indicate that these small molecule compounds can directly bind to IFI27, PKM, and RSAD2, suggesting that they may exert effects by directly targeting key factors of mitochondrial metabolism. However, the therapeutic effects and safety of these candidate drugs in PC animal models and patients still need further validation, and their molecular mechanisms of action also require further exploration.

To further elucidate the cellular heterogeneity of the PC tissue microenvironment and to explore the expression characteristics of key genes in different cellular subpopulations, this study conducted an in-depth analysis of single-cell transcriptomic data from PC. We discovered that PC tissue is primarily composed of various cell types, including fibroblasts, endothelial cells, immune cells, and others, reflecting the complexity of the PC microenvironment. Among these, fibroblasts are the main component of the PC microenvironment and play a crucial role in tumor progression. Studies have shown that fibroblasts can promote PC cell proliferation, invasion, and metastasis by remodeling the extracellular matrix and secreting cytokines [51]. This study further revealed that key genes related to mitochondrial metabolism are significantly upregulated in PC fibroblasts, suggesting that metabolic reprogramming of fibroblasts may be an important mechanism driving tumor progression. Studies have indicated that cancer cells can promote endothelial cell proliferation, migration, and angiogenesis by secreting pro-angiogenic factors such as VEGF, providing nutrients and oxygen for tumor growth [52]. In addition, endothelial cells can mediate immune cell infiltration and inflammatory responses by expressing adhesion molecules and chemokines, affecting the immune status of the tumor microenvironment [53]. Furthermore, we found that IFI27, PKM, and RSAD2 are upregulated in expression across multiple cell types in PC tissue, particularly enriched in fibroblasts and endocrine cells. Endocrine cells are an important component of pancreatic tissue, responsible for secreting hormones such as insulin, and they participate in PC progression [54]. This study is the first to reveal abnormal expression of mitochondrial metabolic genes in endocrine cells at the single-cell level, suggesting that metabolic reprogramming of endocrine cells may be involved in the development of PC. Subsequent studies need to further analyze the molecular mechanisms of endocrine cell metabolic reprogramming and its causal relationship with the development of PC, which may provide new insights for endocrine therapy.

Several limitations warrant explicit acknowledgment. The present study is entirely retrospective and draws on publicly available transcriptomic datasets. The associations reported here—between the three key genes and prognosis, immune infiltration, or candidate drugs—are correlative in nature, and causality cannot be inferred. Immune-cell proportions were estimated by CIBERSORT deconvolution rather than measured directly, and such estimates are known to be sensitive to the choice of reference signature matrix and to the heterogeneous composition of bulk tissue; the gene–immune-cell correlations should therefore be treated as hypothesis-generating rather than confirmatory. The ceRNA and TF regulatory networks were constructed from sequence-based prediction databases that carry non-trivial false-positive rates. Although the expression of the predicted ceRNA and TF regulators was verified in publicly available datasets, the causal regulatory relationships within these networks, together with the proposed drug-target mechanisms, are based on computational inference and still require dedicated experimental validation. Perhaps most importantly, the dual biology of these genes leaves room for alternative readings. The elevated expression of the interferon-stimulated genes IFI27 and RSAD2, for instance, could equally reflect a reactive but ultimately futile anti-tumor immune response rather than a primarily oncogenic program—a distinction the existing literature on interferon signaling in cancer has not fully resolved. We have tried to hold these competing interpretations in tension rather than collapsing them prematurely into a single mechanistic narrative.

On the translational side, the three-gene panel (IFI27, PKM, and RSAD2) achieved a combined AUC of 0.892 in discriminating tumor from normal tissue, a performance level that, if reproduced in prospective cohorts, could support development of a focused RT-qPCR or immunohistochemistry assay for cytologically ambiguous pancreatic lesions where conventional pathology remains equivocal. The consistent association of high expression across all three genes with shorter overall survival lends the nomogram some practical appeal as a post-operative stratification tool, though its clinical utility would need to be evaluated against existing staging criteria before any change to adjuvant therapy decisions could be contemplated. What we find more immediately actionable is the mechanistic convergence the data point toward: PKM-driven glycolytic reprogramming and an immunosuppressed microenvironment—characterized by M2-macrophage enrichment alongside apparent CD8 ⁺ T-cell and NK-cell exclusion—co-occur in the same tumors, and this co-occurrence offers a concrete rationale for combination strategies pairing PKM2-directed or glycolysis-targeting agents with macrophage-repolarizing or checkpoint-based immunotherapy, particularly in IFI27/RSAD2-high disease. Whether this mechanistic logic translates into therapeutic benefit is an open question, but it is one that can be framed as a testable hypothesis and addressed in experimental models without waiting for the bioinformatic signature itself to be fully validated.

This study employed a multi-omics integrative analysis strategy to reveal the molecular pathogenesis of PC from multiple levels, including gene expression, immune microenvironment, and cellular heterogeneity. Future studies could use in vitro and in vivo functional experiments to deeply analyze the roles of IFI27, PKM, and RSAD2 in PC metabolic reprogramming. Additionally, animal models and clinical trials need to be conducted to evaluate the efficacy and safety of candidate drugs and optimize dosing regimens. Lastly, since tumor metabolic reprogramming involves multiple metabolic pathways and regulatory factors, subsequent studies could further integrate metabolomics and proteomics data to construct a more comprehensive and precise PC metabolic regulatory network, providing a theoretical basis and drug screening platform for metabolic therapy.

In summary, this study, starting from single-cell and overall levels, revealed the key roles of IFI27, PKM, and RSAD2 in PC mitochondrial metabolic reprogramming and tumor progression, constructed a multi-level regulatory network of key genes, and proposed potential drug intervention strategies. These findings deepen the understanding of PC metabolic adaptation mechanisms and provide new insights for the diagnosis and treatment of PC in the era of precision medicine. Future studies need to conduct more basic and clinical research to promote the integration of metabolomics and multi-omics technologies, ultimately achieving the goals of early diagnosis and precise treatment of PC.

## 5 Conclusion

This study integrates single-cell and bulk RNA sequencing to investigate mitochondrial metabolic reprogramming in PC. Through WGCNA and machine learning algorithms, we identified three key genes—IFI27, PKM, and RSAD2—that are significantly upregulated in PC and associated with poor prognosis. These genes drive PC progression through metabolic reprogramming, immune evasion, and oncogenic signaling. They are enriched in glycolysis and carbon metabolism pathways, promote immunosuppression by recruiting M2 macrophages while excluding CD8 + T cells and NK cells, and show specific expression in fibroblasts and endocrine cells. The nomogram model demonstrated strong diagnostic capability (AUC = 0.892), and potential therapeutic compounds including amlexanox and ademetionine were identified through drug prediction. In summary, IFI27, PKM, and RSAD2 represent critical drivers of PC through convergent metabolic and immunological mechanisms. These findings provide promising diagnostic biomarkers and therapeutic targets for PC, warranting further experimental validation and clinical translation.
